# Changes in choroidal area after intraocular pressure reduction following trabeculectomy

**DOI:** 10.1371/journal.pone.0201973

**Published:** 2018-08-22

**Authors:** Hirokazu Kojima, Kazuyuki Hirooka, Eri Nitta, Kaori Ukegawa, Shozo Sonoda, Taiji Sakamoto

**Affiliations:** 1 Department of Ophthalmology, Kagawa University Faculty of Medicine, 1750–1 Ikenobe, Miki, Kagawa, Japan; 2 Department of Ophthalmology, Kagoshima University Graduate School of Medical and Dental Sciences, Kagoshima, Japan; Bascom Palmer Eye Institute, UNITED STATES

## Abstract

**Purpose:**

To investigate changes of the macular and peripapillary choroidal areas after trabeculectomy.

**Methods:**

This prospective and interventional study examined 74 eyes of 74 patients with glaucoma uncontrolled by medical therapy. Enhanced depth imaging optical coherence tomography (EDI-OCT) recorded macular and peripapillary choroidal images at 1 day before trabeculectomy and at 2 weeks after surgery. The Niblack method was used to covert luminal and interstitial areas to binary images.

**Results:**

At baseline, the mean intraocular pressure (IOP) was 17.6±6.3 mmHg, while it was 6.5±2.9 mmHg after trabeculectomy (*P* < 0.001). Increases were observed for the macular choroidal area after the surgery, with the total area increasing from 317,853±95,728 μm^2^ to 368,597±104,393 μm^2^, while the luminal area increased from 210,355±73,650 μm^2^ to 249,464±77,195 μm^2^, and the interstitial area increased from 107,498±27,613 μm^2^ to 119,133±31,811 μm^2^ (all *P* < 0.001). Increases were also observed after the surgery for the peripapillary choroidal area, with the total area increasing from 1,629,440±460,429 μm^2^ to 1,974,289±500,496 μm^2^, while the luminal area increased from 920,141±328,690 μm^2^ to 1,179,843±357,601 μm^2^, and the interstitial area increased from 709,299±153,179 μm^2^ to 794,446±169,029 μm^2^ (all *P* < 0.001). There was a significant increase in the ratio of the luminal to choroidal area in the macular area (67.2%) and in the peripapillary area (59.1%). Factors associated with the changes in the peripapillary choroidal area included decreases in the diastolic blood pressure and IOP.

**Conclusions:**

A reduction in the IOP after trabeculectomy led to increases in the macular and peripapillary choroidal areas. Observed changes in the choroidal area after trabeculectomy are primarily due to increases in the luminal areas.

## Introduction

Vascular factors are known to be associated with the development of glaucomatous damage, with blood to the prelaminar, anterior lamina and retrolaminar regions of the optic nerve disc supplied in part by the peripapillary choroid.[1,2] Use of the noninvasive imaging method, optical coherence tomography (OCT), makes it possible to obtain micrometer resolution of in situ cross sections of the retina and choroid.[3] Several studies have examined the choroidal thickness in eyes using enhanced depth imaging OCT (EDI-OCT) and reported finding a thinner thickness in glaucoma compared to healthy subjects.[4–7] In contrast, other studies have reported finding no difference in the choroidal thickness between normal and glaucoma patients.[8–10]

One of the most commonly performed filtration surgeries for reducing the intraocular pressure (IOP) in glaucoma is trabeculectomy. Several investigations have examined the reductions in the IOP that occur after trabeculectomy and reported finding increases in the subfoveal and peripapillary choroidal thicknesses in primary open-angle glaucoma (POAG) and in primary angle closure glaucoma (PACG).[11–13] However, the question that needs to be answered is not only how are these changes able to occur in the choroid thickness, but also, which structures are actually involved in the change? However, in order to answer these questions, a morphometric analysis of the choroid needs to be undertaken. Another issue that needs to be considered when performing choroidal thickness measurements is whether or not preselected positions are used during the procedure. Sonoda et al.[14–16] used the ImageJ open access software to create a new method for differentiating and quantifying the choroidal lumens from the stroma. When using EDI-OCT, images with hyporeflective areas represent the luminal or fluid-filled areas, while hyperreflective areas represent the stromal areas.[17,18]

The purpose of our current study was to evaluate the acute IOP reduction in glaucoma patients after trabeculectomy using EDI-OCT and then evaluate the macular and peripapillary choroidal area changes. In addition, we also investigated any possible association between the postoperative changes of the choroidal area and the IOP.

## Materials and methods

### Subjects

Eligible patients examined between July 2016 and March 2017 at Kagawa University Hospital received a detailed explanation of the study. Written informed consent was provided by all enrolled subjects in accordance with the principles outlined in the Declaration of Helsinki. The Kagawa University Faculty of Medicine Institutional Review Board approved the study protocol.

Glaucoma patients ranging in age between 22 and 88 years old who had uncontrolled IOP while taking maximally tolerated medication were enrolled in the study. All study subjects underwent examinations that included visual acuity, refraction, central and peripheral fields, slit lamp, and gonioscopy. One surgeon (KH) performed the fornix-based trabeculectomy in all of the patients. To be included in the study, patients had to have a spherical refraction within ± 6.0 diopters (D) and a cylinder within ± 2.0 D. Subjects were excluded if they had any history of retinal diseases (e.g., diabetic retinopathy, macular degeneration, retinal detachment), had undergone previous laser therapy, had poor image quality due to unstable fixation, or if they had severe cataract. Subjects were also excluded if there was a previous treatment history with medications that are known to affect retinal thickness (intravitreal anti-VEGF therapy). The same investigator performed the EDI-OCT examinations in all of the cases.

### EDI-OCT

Macular or peripapillary choroidal images were obtained at 1 day before and 2 weeks after surgery using the Heidelberg Spectralis (Heidelberg Engineering, Heidelberg, Germany) with the EDI-OCT technique. All measurements were performed between 1300–1500 hours. Macular region scans were performed using seven horizontal lines of 30 × 10° through the center of the fovea. A 360°, 3.4 mm diameter circle scan centered on the optic disc was used to scan the peripapillary region. The best quality image from at least three scans was chosen for the subsequent analysis. Choroidal thickness was defined as the area that occurred between the outer portion of the hyperreflective line that corresponded to the retinal pigment epithelium (RPE) and the inner surface of the sclera.

### Binarization of the choroid EDI-OCT images

**Fig 1:**
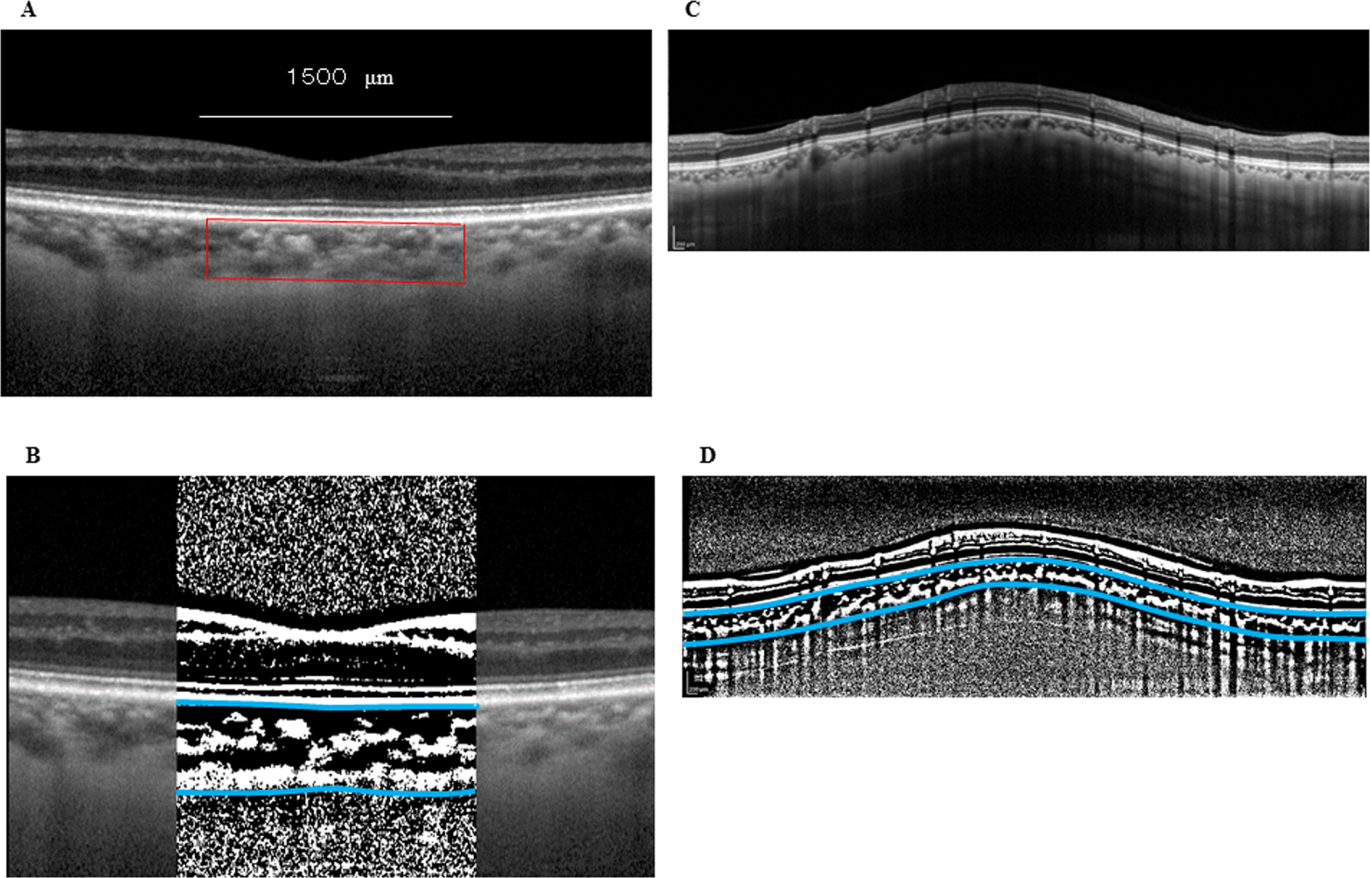
Enhanced depth imaging OCT image and converted binary image of the eye of a glaucoma patient. The EDI-OCT images in the macular area (A) or the peripapillary area (C) were converted to binary images (B, D) using the ImageJ software. The luminal area (dark area) and the interstitial area are seen. The area between the blues lines indicates the measurement area of the choroid.

After EDI-OCT images were recorded, the best images were masked and then displayed on a computer screen. One of the authors (HK) then evaluated each of the images. Using a previously described modified Niblack method,[14] the choroidal area in each of the EDI-OCT images underwent binarization. Briefly, ImageJ (version 1.47, NIH, Bethesda, MD) was used to first analyze the EDI-OCT image. The analysis examined an area of the macular choroid that was 1,500 μm wide and extended vertically (Fig 1A and 1B). This included a 1.7 mm area that was located around the optic nerve disc center (Fig 1C and 1D) and spanned from the retinal pigment epithelium to the chorioscleral border. The ImageJ ROI Manager determined the area to be analyzed. After using the Oval Selection Tool on the ImageJ tool bar to randomly select 3 choroidal vessels with lumens > 100 μm, the reflectivities of these lumens were then averaged. In order to reduce the noise in the OCT image, the average reflectivity was set as the minimum value. Subsequently, the Niblack Auto Local Threshold converted and adjusted the image to 8 bits, with the binarized image then converted to a RGB image once again. The conversions were necessary due to the technical requirements for the binarization procedures and the automated calculation by Image J. The Threshold Tool was used to determine the hyporeflective area, with the dark pixels defined as hyporeflective areas, and the light pixels defined as the hyperreflective areas. The automatic calculation of the hyperreflective and hyporeflective areas was performed after adding the data on the relationship between the distance on the fundus and the pitch of the pixels in the EDI-OCT images, which is dependent on the axial length.

### Statistical analysis

All statistical analyses were performed using SPSS for Windows (SPSS Inc., Chicago, IL). Preoperative and postoperative values were compared by a paired *t*-test. Spearman’s correlation coefficient was used to assess the correlation between changes in the choroidal area, and the correlations among the choroidal area, systolic blood pressure (SBP), diastolic blood pressure (DBP), IOP, age, ocular perfusion pressure (OPP), and axial length. The OPP was defined as, OPP = 2/3[DBP + 1/3(SBP–DBP)]–IOP. Each of the explanatory variables were determined by the univariate analysis. For the multivariate analysis, the choroidal area was defined as a dependent parameter, with four other parameters selected by the univariate analysis and the choroidal area defined as the independent parameters. *P* < 0.05 was considered statistically significant. All statistical values are presented as the mean ± standard deviation (SD).

## Results

This study examined 74 eyes of 74 patients. Clinical characteristics for the enrolled subjects are listed in Table 1.

**Table 1:** Demographic and clinical data of the patients.

Age (years)	67.7±5.3
Gender (M/F)	32/42
Glaucoma type	
Primary open-angle glaucoma	31
Normal-tension glaucoma	22
Secondary glaucoma	8
Exfoliation glaucoma	7
primary angle-closure glaucoma	4
Developmental glaucoma	2

M; male, F; female

After trabeculectomy, mean IOP decreased from 17.6±6.3 mmHg to 6.5±2.9 mmHg (*P* < 0.001), while the mean OPP increased from 45.3±10.0 mmHg to 54.4±10.4 mmHg (*P* < 0.001; Table 2). Axial length decreased from 24.5±1.7 mm before surgery to 23.9±3.3 mm after surgery (*P* = 0.036; Table 2).

**Table 2:** IOP, BP, OPP and axial length before and after trabeculectomy.

	Before	After	*P* value
IOP (mmHg)	17.6±6.3	6.5±2.9	<0.001
BP (mmHg)			
Systolic	127.7±19.0	124.8±20.2	0.22
Diastolic	77.7±13.2	74.6±13.8	0.049
OPP (mmHg)	45.3±10.0	54.4±10.4	<0.001
Axial length (mm)	24.5±1.7	23.9±3.3	0.036

IOP; intraocular pressure, BP; blood pressure

OPP; ocular perfusion pressure

**Fig 2:**
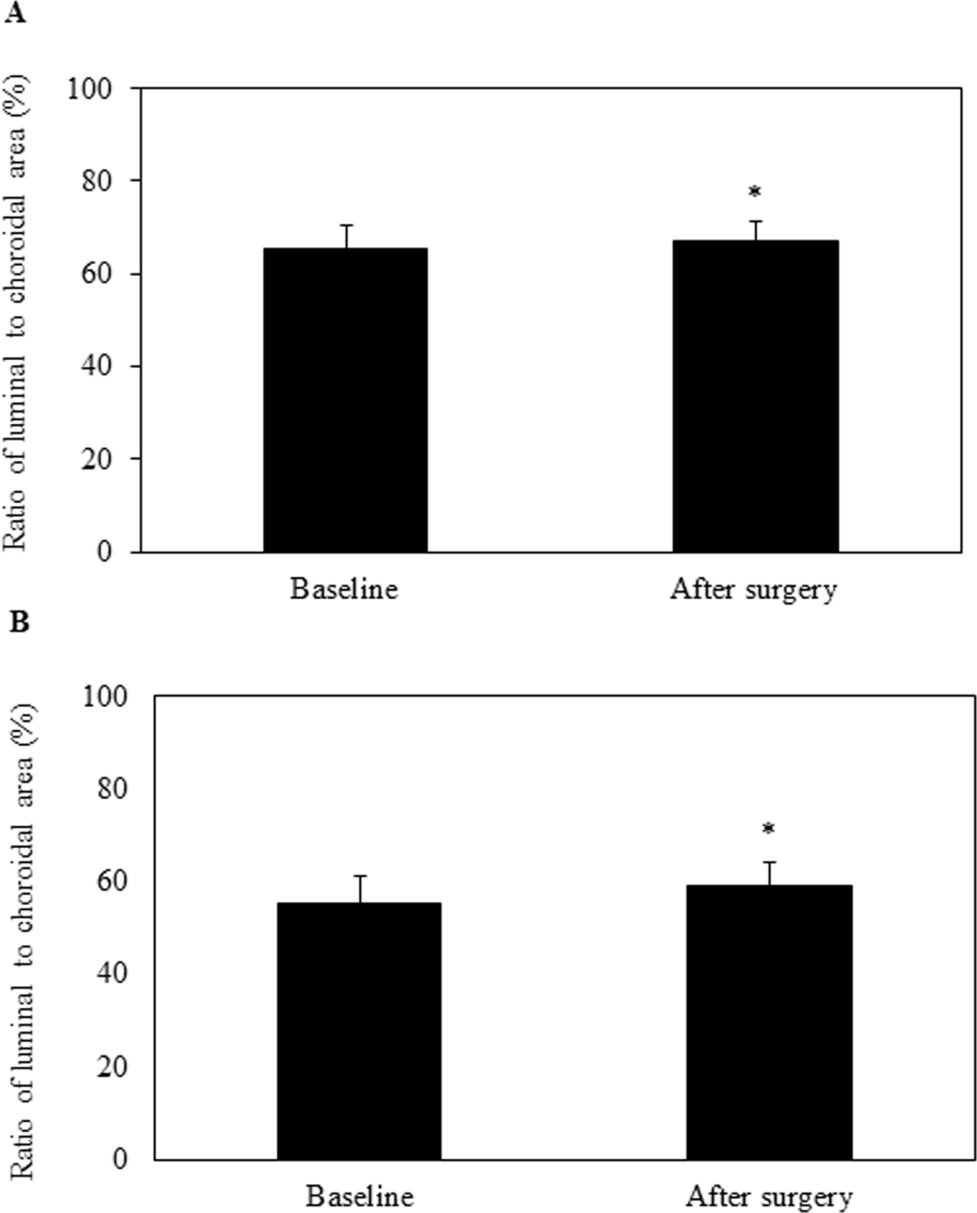
**Ratio of the luminal to the choroidal area for the OCT image after trabeculectomy in the macular (A) and peripapillary (B) region.** At 2 weeks after the trabeculectomy, there was a significant increase in not only the choroidal area but also the ratio of the luminal area to the choroidal area.

Increases were observed for all of the macular choroidal areas after surgery, with the total area increasing from 317,853±95,728 μm^2^ to 368,597±104,393 μm^2^, the luminal area from 210,355±73,650 μm^2^ to 249,464±77,195 μm^2^, and the interstitial area from 107,498±27,613 μm^2^ to 119,133±31,811 μm^2^ (all *P* < 0.001; Table 3). Increases were observed for all of the peripapillary choroidal areas, with the total area increasing from 1,629,440±460,429 μm^2^ to 1,974,289±500,496 μm^2^, the luminal area from 920,141±328,690 μm^2^ to 1,179,843±357,601 μm^2^, and the interstitial areas from 709,299±153,179 μm^2^ to 794,446±169,029 μm^2^ (all *P* < 0.001; Table 3). After surgery, the ratios of the luminal to the interstitial area for the macula and peripapillary were 67.2% and 59.1%, which was significantly larger than that observed before surgery (*P* < 0.001) (Fig 2).

**Table 3:** Choroidal area on EDI-OCT images before and after surgery.

		Macula				Peripapilla		
	Before		After	*P* value	Before		After	*P* value
Total area (μm^2^)	317,853±95,728		368,597±104,393	<0.001	1,629,440±460,429		1,974,289±500,496	<0.001
Luminal area (μm^2^)	210,355±73,650		249,464±77,195	<0.001	920,141±328,690		1,179,843±357,601	<0.001
Interstitial area (μm^2^)	107,498±27,613		119,133±31,811	<0.001	709,299±153,179		794,446±169,029	<0.001

There was a negative correlation between the magnitude of change for the macular choroidal area and the magnitude of change for the axial length (r = -0.33, *P* < 0.001; Table 4). In addition, there was also a negative correlation between the magnitude of the change for the peripapillary choroidal area and the magnitude of the IOP reduction (r = -0.42, *P* < 0.001; Table 5). However, no correlation was observed between the magnitude of the change for the macular choroidal area and the magnitude of the IOP reduction (r = 0.15, *P* = 0.19).

**Table 4:** Pearson's correlation between magnitude change of macular choroidal area and each factor.

	r	*P* value
Age	-0.05	0.68
Changes in SBP	-0.07	0.55
Changes in DBP	-0.16	0.16
Changes in IOP	-0.15	0.19
Changes in OPP	-0.03	0.83
Changes in AL	-0.33	<0.001

SBP: Systolic blood pressure, DBP: Diastolic blood pressure

IOP: Intraocular pressure, OPP: Ocular perfusion pressure

AL: Axial length

**Table 5:** Pearson's correlation between magnitude change of peripapillary choroidal area and each factor.

	r	*P* value
Age	0.19	0.11
Changes in SBP	-0.07	0.57
Changes in DBP	-0.19	0.11
Changes in IOP	-0.42	<0.001
Changes in OPP	0.11	0.34
Changes in AL	-0.17	0.16

SBP: Systolic blood pressure, DBP: Diastolic blood pressure

IOP: Intraocular pressure,OPP: Ocular perfusion pressure

AL: Axial length

We further investigated the factors that might influence the increases observed in the macular choroidal area. As seen in Table 6, the univariate and multivariate analyses for each parameter showed there were no significant correlations with the changes observed in the macular choroidal area. However, our analyses did show that the changes in the DBP and the IOP were significantly associated with the changes in the peripapillary choroidal area (Table 7).

**Table 6:** Univariate and multivariate analysis of associations changes in subfoveal choroidal area and ocular and general parameters.

	Univariate analysis		Multivariate analysis	
	r	*P* value	β	*P* value
Choroidal area	0.12	0.32	-0.12	0.29
Age	0.05	0.68		
SBP	0.02	0.89		
Changes in SBP	0.07	0.55		
DBP	0.01	0.93		
Changes in DBP	0.16	0.16	-0.25	0.06
IOP	0.12	0.31	-0.22	0.43
Changes in IOP	0.15	0.19	-0.31	0.25
OPP	0.09	0.45		
Changes in OPP	0.03	0.83		
Axial length	0.15	0.20	0.17	0.24

SBP: Systolic blood pressure, DBP: Diastolic blood pressure, IOP: Intraocular pressure

OPP: Ocular perfusion pressure, AL: Axial length

**Table 7:** Univariate and multivariate analysis of associations changes in peripapillary choroidal area and ocular and general parameters.

	Univariate analysis		Multivariate analysis	
	r	*P* value	β	*P* value
Choroidal area	0.15	0.20	-0.02	0.85
Age	0.19	0.11	0.19	0.12
SBP	0.15	0.19		
Changes in SBP	0.07	0.57		
DBP	0.16	0.17		
Changes in DBP	1.19	0.11	-0.25	0.02
IOP	0.29	0.01	-0.39	0.11
Changes in IOP	0.42	<0.001	-0.74	<0.001
OPP	0.03	0.83		
Changes in OPP	0.11	0.34		
Axial length	0.10	0.38		

SBP: Systolic blood pressure, DBP: Diastolic blood pressure, IOP: Intraocular pressure

OPP: Ocular perfusion pressure, AL: Axial length

Thirty patients were investigated changes of the macular and peripapillary choroidal areas at 1 year after surgery. Increases were observed for the macular choroidal area at 1 year after surgery, with the total area increasing from 317,735±77,380 μm^2^ to 338,120±90,700 μm^2^ (*P* = 0.03) and the interstitial area increased from 108,598±27,613 μm^2^ to 119,172±31,496 μm^2^ (*P* = 0.01) (Table 8). Increases were also observed at 1 year after surgery for the peripapillary choroidal area, with the total area increasing from 1,557,487±431,798 μm^2^ to 1,650,253±466,672 μm^2^ (*P* = 0.03) and the interstitial area increased from 689,891±149,476 μm^2^ to 751,816±162,457 μm^2^ (*P* = 0.001) (Table 8). However, there was no difference in the macular and peripapillary luminal areas at 1 year after surgery.

**Table 8:** Choroidal area on EDI-OCT images before and 1 year after surgery.

		Macula	(n = 30)			Peripapilla	(n = 30)	
	Before		After	*P* value	Before		After	*P* value
Total area (μm^2^)	317,735±77,380		338,120±90,700	0.03	1,557,487±431,798		1,650,253±466,672	0.03
Luminal area (μm^2^)	209,137±56,767		218,948±61,424	0.15	867,596±301,209		898,437±312,174	0.28
Interstitial area (μm^2^)	108,598±24,502		119,172±31,495	0.01	689,891±149,476		751,816±162,457	0.001

## Discussion

The present study investigated changes in the macular and peripapillary choroidal areas and in the IOP in glaucoma eyes before and after trabeculectomy. After trabeculectomy, significant increases were observed in both areas. In addition, we also found that the increases in the choroidal areas for both the macula and peripapillary occurred in conjunction with a decreasing IOP in both the large choroidal vessels and the interstitium of the choroid.

Previous studies have also reported finding that the choroidal thickness changes were associated with decreases in the IOP after trabeculectomy.[11–13] For example, Kara et al.[11] found changes after measuring the choroidal thickness at the fovea, 1000 μ nasal to the fovea, and 1000 μ temporal to the fovea, while Chen et al.[12] measured the choroidal thickness at the fovea and at 1 and 3 mm superior, inferior, nasal, and temporal from the fovea. Kadziauskiene et al.[13] also measured choroidal thickness at the fovea and at 1.7 mm superior, temporal, inferior and nasal to the optic disc center. However, in all of these studies, the authors only focused on a few separate linear measurements. In contrast, choroidal measurements in our current study were obtained using a macular choroidal area that was 1,500 μm wide and which extended vertically to 1.7 mm around to the optic nerve disc center. Thus, the increased size of our measurements made it possible to obtain a much larger amount of information from the choroid than the previous studies.

From a clinical aspect, we examined the effect of a reduction in the IOP after a trabeculectomy on the luminal and interstitial areas of the choroid. Previous studies have reported finding that increases in choroidal thickness at lower IOPs were associated with approximately equal increases in both their vessels and the interstitium.[19] While our findings indicated that there was an increase in size in both areas, the increase was greater in the luminal versus the interstitial area after trabeculectomy in both the macular and peripapillary choroidal areas. The ratios of the luminal to the choroidal area in the macular and peripapillary areas were 65.4% and 55.4% at baseline, with increases to 67.2% and 59.1% after trabeculectomy, respectively. Since we found an increase in the luminal areas, it is reasonable to assume that there was also an increase in the number of vessels and/or the diameter of vessels. In addition, it has been previously reported that increases in the ocular blood flow after trabeculectomy can also contribute to choroidal thickening.[11,20] Therefore, it is possible that the increase in the diameter of vessels was due to an increase in the ocular blood flow after the trabeculectomy.

Although we found that no factor was significantly associated with the macular choroidal area, the magnitude of change in the macular choroidal area appeared to be correlated with the magnitude of the change in the axial length. Previous studies have reported finding that decreases in the axial length were associated with a decrease in IOP after trabeculectomy.[21,22] Furthermore, other investigators have reported finding a significant correlation between postoperative changes in the choroidal thickness and shortening of the axial length.[11,13] In our study, we found there was a significant increase in the peripapillary choroidal area after trabeculectomy, with this increase correlated with the observed changes in the IOP. Multiple linear regression analysis also demonstrated that the peripapillary choroidal area was significantly associated with changes in the IOP and DBP. Kadziauskiene et al.[13] examined changes in the average, inferior, superior and temporal peripapillary choroidal thicknesses and reported finding a correlation with the magnitude of the reduction in the IOP. There may be several mechanisms that could be responsible for the increases in the choroidal area after trabeculectomy. For example, the reduction in the IOP could directly affect the choroid, as the force of the decreased IOP on the choroid is reduced. Previous studies have demonstrated that increases in ocular blood flow can contribute to choroidal thickening.[11,20] Thus, the decrease in the IOP that is observed after trabeculectomy might be responsible for directly causing the expansion of the macular choroid area, with the decrease in the IOP after trabeculectomy potentially leading to an increase in the ocular blood flow to the choroid, which in turn then causes an increased peripapillary choroid area. After trabeculectomy, the ratio of the luminal to choroidal area increased 102.8% and 106.7% in the macular and the peripapillary choroidal areas, respectively. However, a further study will need to be undertaken in order to definitively prove our hypothesis.

There were some limitations for our current study. First, since there was no OCT software available for performing automated segmentation, all the identifications of Bruch’s membrane and the inner scleral border were manually conducted. Another limitation is that we only investigated the changes that occurred at 2 weeks postoperative. Therefore, the exact amount of time that the changes in choroidal area persist after the trabeculectomy remains unknown. Further long-term research will need to be undertaken in order to establish which of the choroidal area changes continue and for what length of time.

## Conclusions

In conclusion, the present study demonstrated that the IOP reduction after trabeculectomy caused an increase in the macular and peripapillary choroidal areas, with this increase primarily due to an increase in the luminal areas. Changes in the DBP and in the IOP were significantly associated with changes in the peripapillary choroidal area.
